# Quantum interference of pulsed time-bin entanglement generated from silicon ring resonator

**DOI:** 10.1038/s41598-024-51311-4

**Published:** 2024-01-11

**Authors:** Takafumi Ono, Yoshiaki Tsujimoto, Kentaro Wakui, Mikio Fujiwara

**Affiliations:** 1 Program in Advanced Materials Science Faculty of Engineering and Design, Kagawa University, 2217-20 Hayashi-cho, Takamatsu, Kagawa 761-0396 Japan; 2 JST, PRESTO, 4-1-8 Honcho, Kawaguchi, Saitama 332-0012 Japan; 3 National Institute of Information and Communications Technology (NICT), Koganei, Tokyo 184-8795 Japan

**Keywords:** Quantum physics, Quantum optics

## Abstract

We demonstrate a pulsed operation of an entangled photon pair source that is based on a silicon ring resonator. Time-bin entangled photon pairs at telecommunication wavelengths are generated via spontaneous four-wave mixing, which is excited by a pulsed pump laser. The entanglement between the generated photon pair is analyzed by using asymmetric Mach–Zehnder interferometers followed by single-photon detectors, resulting in non-classical interference with a visibility exceeding a classical limit. The reason for the degradation of the interference visibility is discussed using the theoretical model with experimental parameters. Our experimental results show successful pulsed generation of entanglement, which represents an important step towards a synchronized quantum network based on silicon photonics.

## Introduction

Integrated photonics plays a crucial role in the realization of photonic quantum information processing with a compact design^1–9^. A silicon-based optical integrated circuit is one of the most promising platforms since it is available on matured CMOS platforms offering dense integration and has the advantage of low optical loss in the telecom band^10,11^. The third-order nonlinearity of silicon allows the generation of photon pairs on-chip via spontaneous four-wave mixing (SFWM), making it possible to integrate both the photon pair source and an optical quantum circuit on a single chip. Several experiments have demonstrated photon pair generation in silicon with various structures such as waveguides^12,13^, disks^14^, and ring resonators^15–18^. In particular, silicon ring resonators offer a strong interaction between pump light and silicon, which allows the generation of photon pairs with less than milliwatts of optical pump power^19,20^. Furthermore, a flexible design of the resonator enables the generation of narrow-band photons down to sub-GHz and frequency multiplexing complying with ITU standards^21^.

In previous experiments employing silicon ring resonators, energy-time entanglement has been generated by using a continuous wave (CW) pump laser^17,18,21,22^, where the photon pairs are continuously produced over the coherence time of the pump light, and time-bin is selected by asymmetric Mach–Zehnder interferometers (AMZI) for the measurement. On the other hand, in advanced protocols such as entanglement swapping toward quantum internet, pulsed operation of the entangled photon pair sources is desirable^23–25^. In a pulsed regime, however, the time-bin is defined between the temporal modes of the sequential pulses, which requires strict stabilization of the interval of the pump pulses and a relative delay of an AMZIs. While the generation of sequential time-bin entanglement has been reported using high-refractive-index glass (Hydex glass)^26^ and $\mathrm {Si_3N_4}$ microring resonators^27^, as far as we know, no experiment has been reported on the generation of pulsed time-bin entangled photon pairs using a silicon-ring platform.

In this study, we for the first time report on the generation of time-bin entanglement using a silicon ring resonator in a pulsed regime. A pair of pump pulses were generated by an AMZI and was used to produce signal and idler photons via SFWM in the silicon ring resonator. The generated signal and idler photons are divided into different spatial modes by frequency filters, and the time-bin entangled state was then analyzed by using another AMZI placed on each arm. We have successfully observed quantum interference with a visibility of about 53% that exceeds the classical limit of 1/3, as derived under the assumption of Werner state^28^. We theoretically analyzed the effect of the instability of the interferometers on the interference visibility. By comparing our theoretical results with the interference visibilities measured for different integration times, we observed good agreement between them. Our result clearly shows the successful generation of a pulsed entangled photon pair based on a silicon-ring resonator and thus is an important step towards a synchronized quantum network using integrated silicon photonics.

## Theory

**Figure 1:**
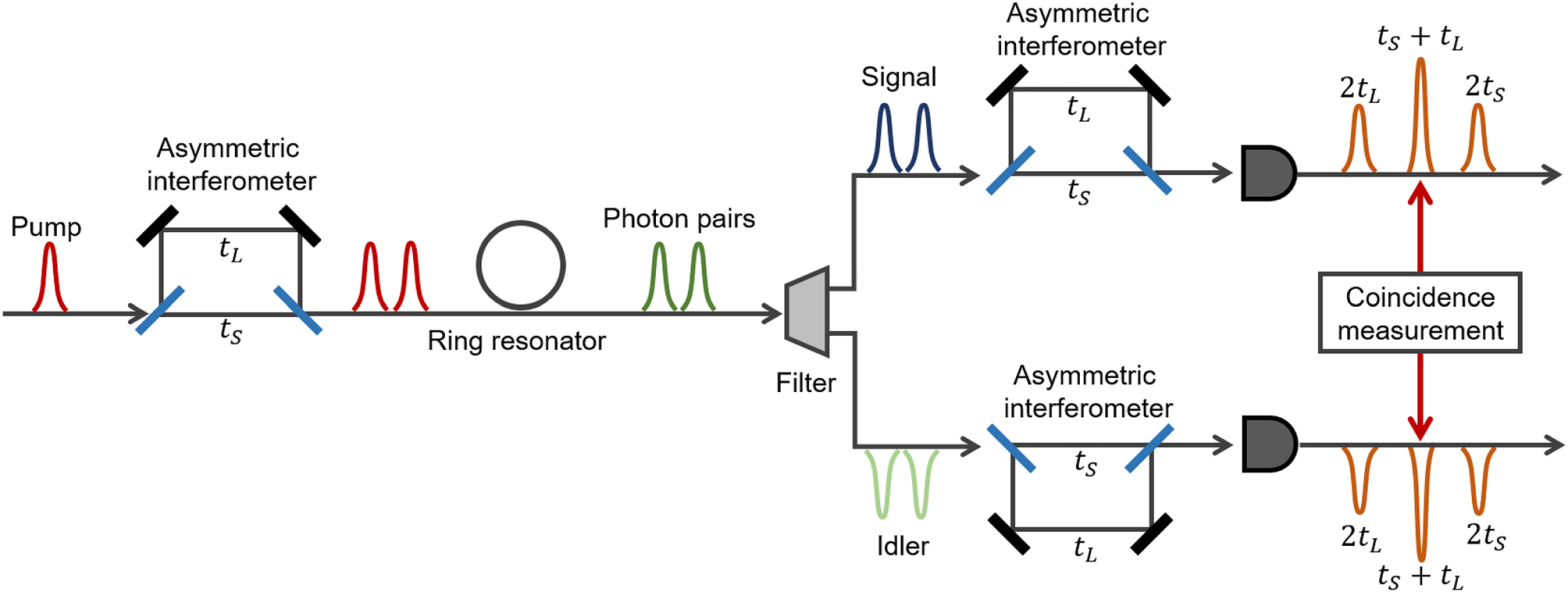
Schematic diagram of the generation and measurement of time-bin entangled states. Initially, a laser pulse is converted into a double pulse after passing through a AMZI. In the ring resonator, spontaneous four-wave mixing is utilized for the conversion of the pump light into signal and idler photons. The generated photon pairs are measured by using a AMZI and a single-photon detector within each arm. In the main text, we define $t_S=0$ and $t_L=\tau$. Coincidences between the center peaks are extracted as events of interest.

Figure 1 shows a schematic diagram illustrating the generation of time-bin entangled states using a pulsed pump light. A double pulse is prepared by injecting a laser pulse into an AMZI, and is then coupled to a silicon ring resonator. By each pump pulse, signal and idler photons are generated via SFWM. Owing to the structure of the ring resonator, the output photons have a comb-like spectrum. We extract a certain frequency combination of the photon pair from the spectrum by using off-chip frequency filters. Assuming that the average photon numbers of the photon pair generated by each pump pulse are equivalent, the two-photon component of the quantum state is given by

1 $$\begin{aligned} | \psi \rangle _{in} \propto (\hat{a}^\dagger _s(t)\hat{a}^\dagger _i(t)+e^{i\theta }\hat{a}^\dagger _s(t+\tau )\hat{a}^\dagger _i(t+\tau ))| vac \rangle , \end{aligned}$$

where $\hat{a}^\dagger _{s(i)}(t)$ is a photon creation operator in the signal (idler) mode at time *t* that satisfies the commutation relation as $[\hat{a}_j(t_1), \hat{a}^\dagger _k(t_2)]=\delta _{jk}\delta (t_1-t_2)$. $\tau$ and $\theta$ are the relative delay and phase added by the AMZI, respectively, and $| vac \rangle$ is a vacuum state. During the experiment, $\tau$ and $\theta$ are fixed.

$| \psi \rangle _{in}$ is evaluated using two AMZIs, more precisely Franson interferometers, with a relative delay of $\tau$. Extracting the events where the signal and idler photons are detected at the same time, we obtain

2 $$\begin{aligned} | \psi \rangle _{out}\propto & {} (\hat{a}^\dagger _s(t)\hat{a}^\dagger _i(t)+e^{i(\phi +\theta )} \hat{a}^\dagger _s(t+2\tau )\hat{a}^\dagger _i(t+2\tau ) \nonumber \\ {}{} & {} +e^{i\theta }\hat{a}^\dagger _s(t+\tau )\hat{a}^\dagger _i(t+\tau )\nonumber \\ {}{} & {} +e^{i\phi }\hat{a}^\dagger _s(t+\tau )\hat{a}^\dagger _i(t+\tau ))| vac \rangle , \end{aligned}$$

where $\phi$ is the sum of the relative phase added by AMZI1 and AMZI2. The last two terms show the quantum interference between the signal and idler photons, which can be extracted by employing coincidence windows at the center peak in the time histogram of D1 and D2, as shown in Fig. 1. The normalized coincidence probability within the coincidence windows is given by

3 $$\begin{aligned} \frac{1}{4}|\langle vac |\hat{a}_s(t+\tau )\hat{a}_i(t+\tau )| \psi \rangle _{out}|^2=\frac{1}{2}(1+\textrm{cos}(\phi -\theta )). \end{aligned}$$

Thus, an interference fringe with visibility of 100% would be observed depending on the relative phase difference $\phi$ of the AMZIs for measurement. We note that if one collects all the coincidence events in Eq. (2), as in the case of energy-time entanglement, the visibility is limited to 50% due to the noninterfering terms.

**Figure 2:**
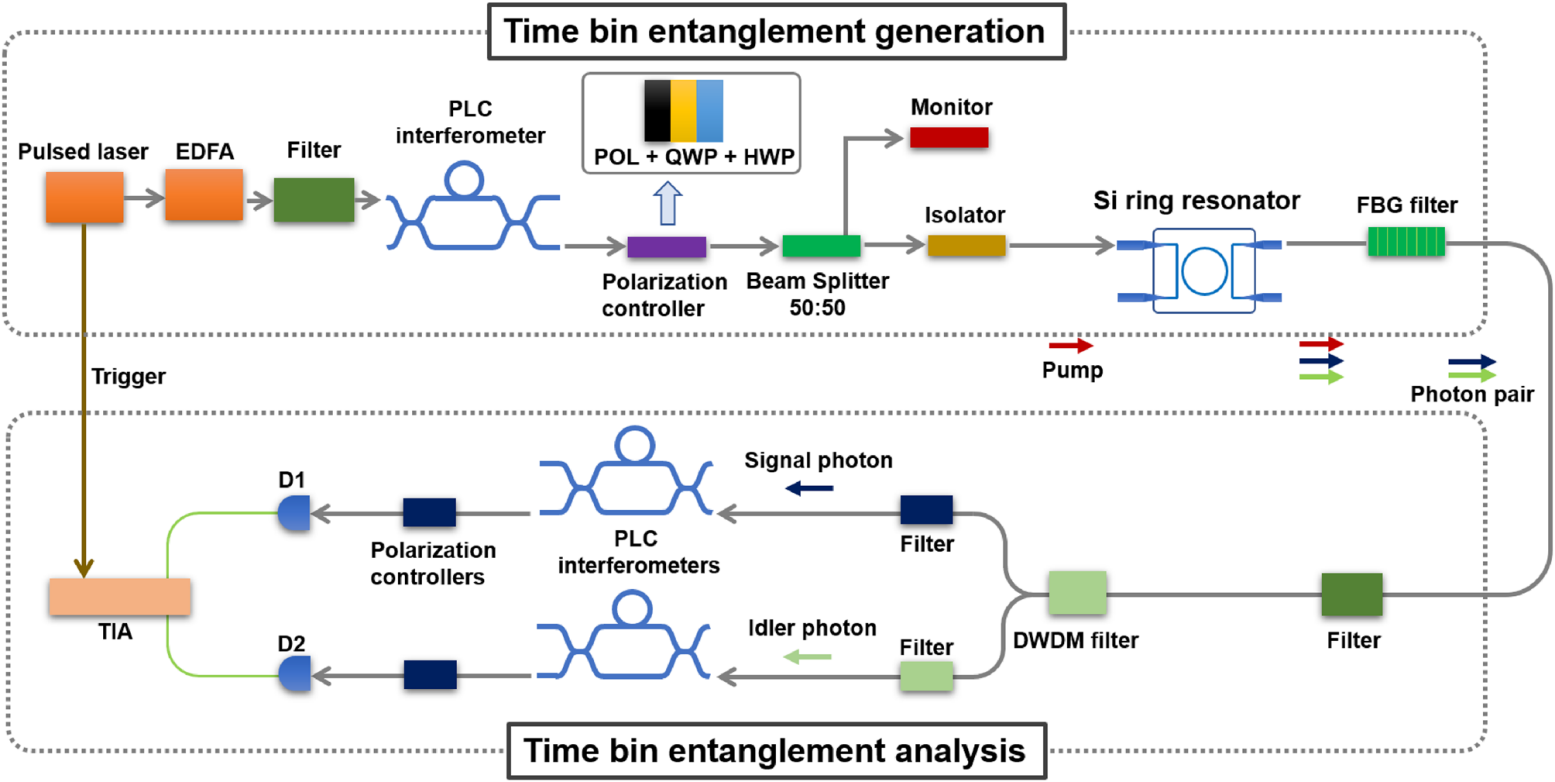
Set-up for the generation and measurement of pulsed time-bin entangle states. The setup consists of two parts: the first part is designed for the generation of the time-bin entangled state, and the second part serves to measure the time-bin entangle state. The electrical signal from the pulsed laser is used to trigger the temporal analysis. The components employed in the experiment include an erbium-doped fiber amplifier (EDFA), a polarizer (POL), a quater wave plate (QWP), a half wave plate (HWP), a fibre Bragg grating (FBG), a wavelength division multiplexing (WDM), and a time interval analyzer (TIA).

## Experiment

Figure 2 shows the experimental setup to generate and analyze time-bin entanglement. We use a pulsed pump laser with a repetition rate of 100 MHz, a center wavelength of 1552.6 nm, and a pulse width of 35 ps. The intensity of the pump laser is amplified by an erbium-doped fiber amplifier (EDFA), and the amplified spontaneous emission is removed by a frequency filter with a bandwidth of 1 nm. The pump pulses are then fed into the Planar Lightwave Circuit (PLC) interferometer, widely utilized as a platform for implementing various optical quantum circuits^3^, to generate a double pulse. The relative delay of the PLC interferometer is 2500 ps, which ensures that the double pulse is sufficiently separated in time. The relative phase of the PLC interferometer is stabilized by a temperature controller. After the PLC interferometer, the double pulse is injected into the straight waveguide of silicon by side coupling via a spot size converter and coupled to a silicon ring resonator through evanescent coupling. The ring resonator has a radius of 10 $\upmu$m and is temperature controlled using Peltier elements. The quality factor of the ring resonator was estimated to be from 15000 to 20000. The free spectral range of the ring resonator was estimated at 12.7 nm. The detail of the ring resonator is given in Refs.^17,18^. Signal and idler photons are generated via SFWM in the silicon ring resonator. The pump light is removed using a fiber-based Bragg grating filter. A pair of the signal and idler photons satisfying energy conservation is extracted by using a frequency-multiplexing filter, where the center wavelengths of each frequency are $1540.1\pm 0.5$ nm and $1565.4 \pm 0.5$ nm, respectively. Each signal and idler photon is analyzed by another PLC interferometer whose relative delay is 2500 ps that is the same as that used to create the double pump pulse. Photons were detected by superconducting single photon detectors (SSPDs)^29^ D1 and D2. The electric signal from the pulsed laser is used as a start signal of the time-to-digital converter (TDC), and the electric signals from the SSPDs are used as stop signals.

**Figure 3:**
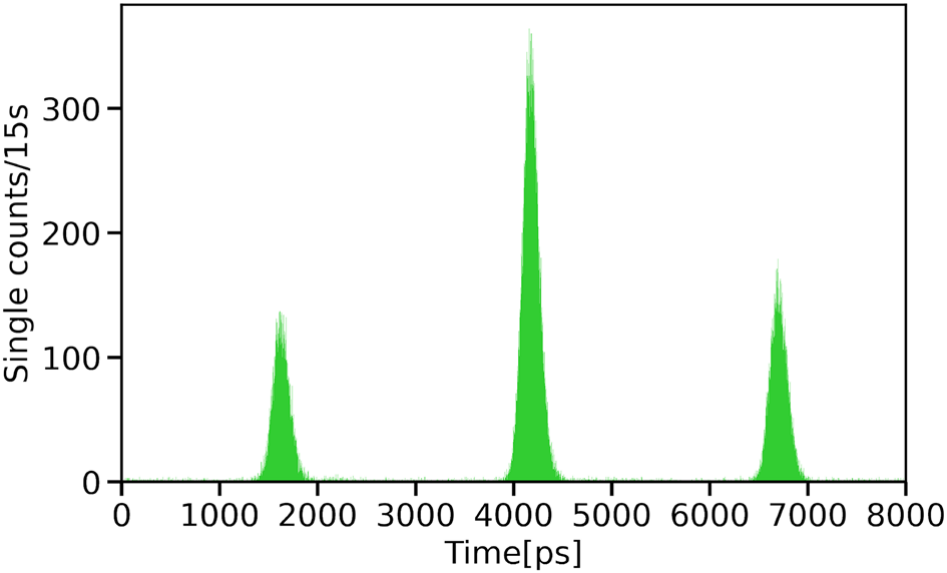
A histogram that displays the temporal distribution of photon arrival times recorded by the D1 detector. Three peaks which corresponds to Eq. (2) were observed.

**Figure 4:**
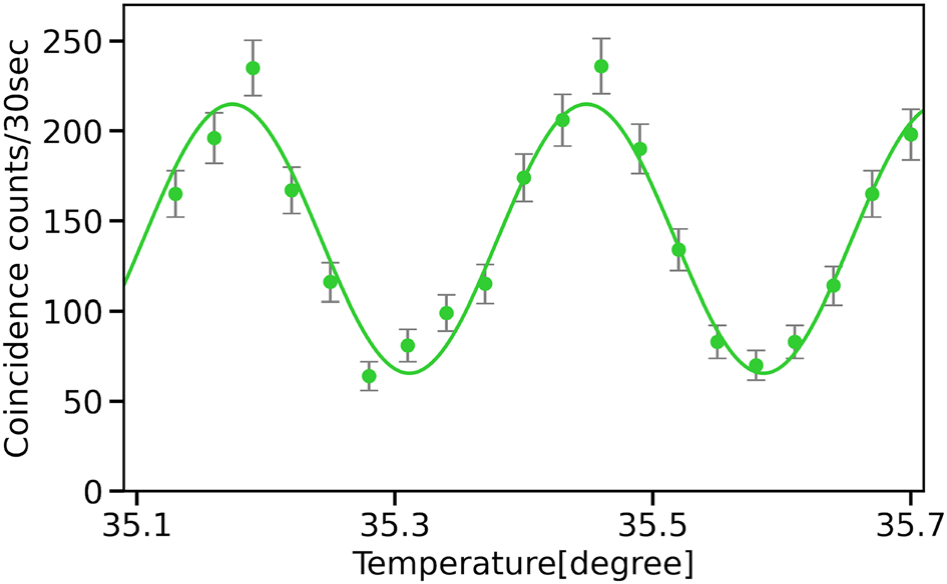
Quantum interference fringes of time-bin entangled states. The vertical axis represents the coincidence counts of the signal and idler photons arriving at a time $t_S + t_L$. The horizontal axis represents the temperature of the PLC interferometer for the idler arm. The error bars are plotted assuming that the number of photon pairs detected is Poisson distributed.

We show the time histograms of D1 in Fig. 3. Three peaks are observed as expected. We employ coincidence windows with a width of 200 ps around the center peaks in the time histograms of D1 and D2. Figure 4 shows the quantum interference between the signal and idler photons. We vary the relative phase of the idler photon with stabilizing that of the signal photon using temperature controllers. As expected from Eq. (3), we have observed interference fringes by varying the relative phase $\phi$. From the fitted curve, the visibility of the interference fringes was estimated to be about $53.3 \pm 3.5$%. This value is larger than the classical limit of 1/3, which is derived assuming Werner state^28^. Therefore, we conclude that we have successfully generated a pulsed time-bin entangled state using a silicon ring resonator.

Finally, we evaluated the spectral purity of the heralded single photons (HSPs). Generation of the spectrally pure HSPs is essential for various applications such as entanglement swapping, which exploits the Hong–Ou–Mandel interference between independently generated HSPs. The spectral purity is determined by measuring the intensity correlation $g^{(2)}(0)$ of the signal (idler) photon, where signal (idler) photon is fed to Hanbury Brown–Twiss (HBT) setup with a half beamsplitter, and the coincidence between each output is recorded. When the signal (idler) photon is in a spectrally single mode, the photon statistics show a super-Poisson distribution, which leads to $g^{(2)}(0)=2$. As the mode number of the signal (idler) photon increases, the photon statistics approaches to a Poisson distribution characterized by $g^{(2)}(0)=1$. The relation between the number of modes and $g^{(2)}(0)$ is approximately given by $g^{(2)}(0) \sim 1+1/n$^30^, where *n* is the number of modes. By performing HBT experiment on the signal photon at 1540 nm, we observed $g^{(2)}(0)=1.59\pm 0.01$ and $n=1.72\pm 0.04$. We see that the HSP is clearly in multi-mode. The reason is considered as follows. It is known that, for obtaining spectrally pure HSPs, the pump bandwidth must be much larger than that of HSPs^30^. In our setup, the bandwidth of the heralded single photon is around 0.09 nm, which is determined by the parameter of the ring resonator. On the other hand, pump bandwidth is also determined by the ring resonator and is also limited by 0.09 nm. In such a situation, the spectral purity cannot be high, even if the spectrally broad pump pulses are used. In a SFWM process in a ring resonator, the signal and idler photons as well as the pump light resonate. Consequently, the bandwidths of the pump light and photons are inevitably same, even when the bandwidth of the pump light is initially larger than that of signal (idler) photons. To increase the purity, one way is to insert another narrow frequency filter in the signal arm. By inserting a frequency filter with a width of 0.05 nm just before the HBT setup, we obtained the spectrally pure photon with $g^{(2)}(0)=1.95\pm 0.08$ and $n=1.05\pm 0.09$. Another way to improve the purity is using temporal filtering with photon detectors with low timing jitter^27,31^.

## Discussion

While the visibility of the quantum interference fringe obtained in this experiment exceeds the classical limit, it is lower than those obtained in the CW-pump regime, as reported in previous studies^18,32^. The degradation in the visibility is mainly attributed to the temperature and mechanical instability of the PLC interferometers used in the experiment. Specifically, we employed the PLC interferometers with a path-length difference of 50 cm, which is longer than those installed in the previous experiments. In particular, since the experiments conducted in this study have PLC interferometers in both the front and back stages of the ring resonator, the influence of phase instability on the visibility seems to be higher than in previous experiments^32^. Here, we discuss the effect of phase instability on the interference visibility in terms of the temperature of the PLC interferometer.

**Figure 5:**
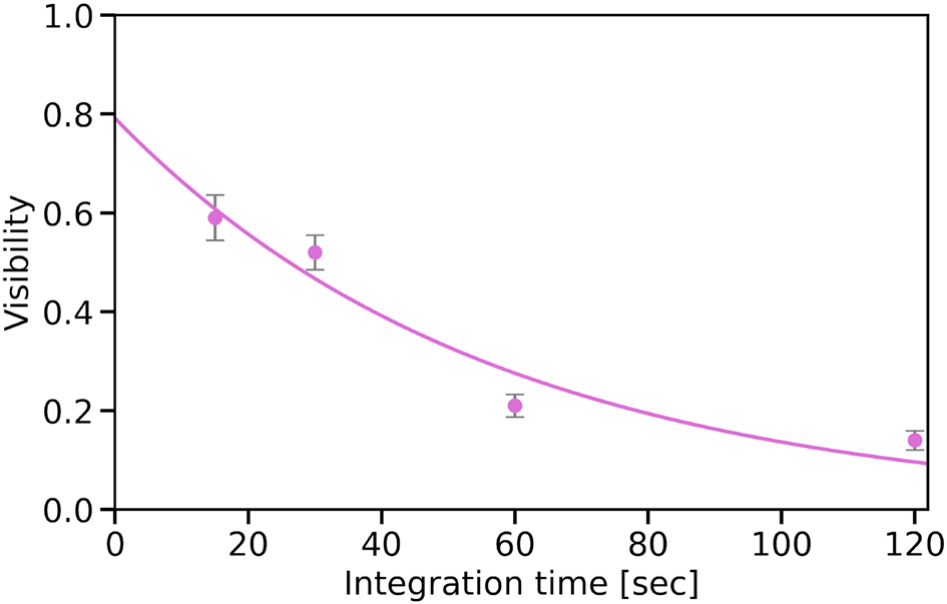
Dependence of the visibility of quantum interference fringes on integration time. The error bars were determined from the raw experimental data, assuming that the fluctuation in the experimental data followed a Poisson distribution. To quantify this, we conducted 3000 Monte Carlo simulations to represent the range of the errors in the visibility obtained from these simulations.

We assume that the phase $\phi$ fluctuates randomly in time *t* due to the instability of the PLC interferometer. The interference fringes, $I(\phi )$, at any given phase $\phi$ can be expressed as

4 $$\begin{aligned} I(\phi ) = \frac{1}{2} \left( 1 + V_{rest.} \cos (\phi ) \right) , \end{aligned}$$

where we introduce $V_{rest.}$ as the residual degradation of visibility that cannot be attributed to the phase instability of the PLC interferometer. The phase drift is then described by the diffusion equation that characterizes the density fluctuations of the phase with a diffusion coefficient of *D*. Specifically, the phase drift is distributed around the center of the true value of $\phi _0$ with a Gaussian function, represented by $g(\phi - \phi _0) = e^{-(\phi - \phi _0)^2/2\Delta \phi ^2}/(\sqrt{2\pi } \Delta \phi )$ where $\Delta \phi = \sqrt{2Dt}$ indicates the degree of phase fluctuation at *t* seconds after setting the phase to $\phi _0$. The average interference fringes at a specific time, denoted by $\left\langle I(\phi _0, t) \right\rangle$, are obtained through the convolution integral between Eq. (4) and $g(\phi - \phi _0)$, as given by

5 $$\begin{aligned} \left\langle I(\phi _0, t) \right\rangle= & {} \int ^{\infty }_{\infty } I(\phi ,t) g(\phi - \phi _0) d\phi \nonumber \\= & {} \frac{1}{2} \left( 1 + V_{rest.}\textrm{e}^{-D t} \cos (\phi _0) \right) , \end{aligned}$$

where $\textrm{e}^{-D t}$ denotes the decay of interference visibility caused by the instability of the PLC interferometer. Figure 5 shows the dependence of interference visibility on integration time. It is observed that the visibility decreases with an increase of integration time. From the fitting, the phase diffusion coefficient was estimated to be $D \approx 0.018$ [rad$^2$/s], and the visibility degradation, which cannot be attributed to the instability of the PLC interferometer, was found to be $V_{rest.} \approx 0.79$. Therefore, reducing the integration time or enhancing the stability of the PLC interferometer can potentially improve the interference visibility up to about 80%. To improve the stability of the interferometer, it is necessary to enhance the accuracy of the temperature controller and reduce mechanical vibrations. One way is to install PLCs with a shorter path length difference. We also noticed that, as indicated in the literature^22,26^, the utilization of a fiber-based Franson interferometer with Faraday mirrors can yield significantly increase visibility, as it is resilient against the polarization fluctuations inside AMZIs. The adoption of such a fiber-type Franson interferometer may lead to further improvements in visibility. On the other hand, the production rate of photon pairs in our experiments is so low that it is difficult to reduce the integration time any further. This is mainly due to the losses incurred in the experimental system, where the transmission rates of the signal and idler photons are estimated to be 0.15% and 0.21%, respectively. These losses include propagation losses in the ring resonator, coupling losses from the optical fiber to the ring resonator and PLC, losses in multiple frequency filters employed between the source and detectors, and quantum efficiencies of the superconducting single photon detectors. In this experiment, we estimated the average number of photons per time bin to be approximately $\mu \approx 0.01$. With a frequency (*f*) of the pulsed light source set at 100 MHz, the theoretical number of photon pairs generated per second would be calculated as $\mu \times f \approx$ 1 MHz, assuming no optical losses. Therefore, addressing these optical losses is essential to achieve a higher count rate.

As shown above, the instability of the PLC interferometer is one of the main reasons for the degradation of interference visibility. On the other hand, the value $V_{rest.} \approx 0.79$ suggests that the remaining approximately 20% is caused by the other factors. In the following, we consider the influence of the multi-pair generation on the interference visibility. We assume that the quantum state of each time-bin is given by the two-mode squeezed vacuum (TMSV) state as $|\psi \rangle = \sum _{n=0}^{\infty } \textrm{sech}^2r\textrm{tanh}^nr | n \rangle _{s} | n \rangle _{i},$ where *r* is the squeezing parameter of the TMSV state and $| n \rangle _{s(i)}$ is *n*-photon Fock state in signal (idler) mode, respectively. To numerically calculate the interference visibility, we borrow the numerical model based on the characteristic function developed in Ref.^33^ by replacing the polarization degree with the temporal degree. From the experimental results, we obtain the experimental parameters as $\eta _s=2.1\times 10^{-3}$, $\eta _i=1.5\times 10^{-3}$, $d=5.0\times 10^{-7}$, and $\mu =1.1\times 10^{-2}$. Here, $\eta _{s(i)}$ is the detection efficiency including the system transmittance of signal (idler) photon, *d* is the dark-count probability within the 500 ps-coincidence window, and $\mu$ is the average photon number of each time-bin which is related to the squeezing parameter as $\textrm{tanh}^2r=\mu /(1+\mu )$. Using these parameters, the degradation of the visibility due to the multi-pair generation is estimated to be 2%, which indicates that the detrimental influence of the multiple pairs is small.

Other possible factors include spatial mode mismatches due to differences in the delay lengths of the PLC interferometers before and after the ring resonator, and nonlinear effects due to two-photon absorption and associated free carrier absorption caused by inputting double pulses into the ring resonator, which will be interesting for future study.

## Conclusions

In conclusion, we have successfully generated pulsed time-bin entanglement using a silicon ring resonator. We observed quantum interference between the signal and idler photons with a visibility of more than 50%. We then analyzed the effect of phase instability in the PLC interferometers on interference visibility. The theoretical model suggests that further improvement of the interference visibility can be expected by developing more stable unbalanced interferometers and/or reducing the integration time of the measurement. In principle, our results indicate the potential for introducing pulsed operation into a silicon-based photon source with time-bin entanglement, utilizing CMOS process technologies. This development represents an important step towards synchronized quantum information processing such as the establishment of a quantum internet based on integrated photonics.

## Data Availability

The datasets used and analysed during the current study available from the corresponding author on reasonable request.
